# Protopia: a protein-protein interaction tool

**DOI:** 10.1186/1471-2105-10-S12-S17

**Published:** 2009-10-15

**Authors:** Alejandro Real-Chicharro, Iván Ruiz-Mostazo, Ismael Navas-Delgado, Amine Kerzazi, Othmane Chniber, Francisca Sánchez-Jiménez, Miguel Ángel Medina, José F Aldana-Montes

**Affiliations:** 1 University of Malaga and "CIBER de Enfermedades Raras, Instituto de Salud Carlos III" (unit 741), Department of Molecular Biology and Biochemistry. Faculty of Sciences, Spain; 2 University of Malaga, Computer Languages and Computing Science Department, Spain

## Abstract

**Background:**

Protein-protein interactions can be considered the basic skeleton for living organism self-organization and homeostasis. Impressive quantities of experimental data are being obtained and computational tools are essential to integrate and to organize this information. This paper presents Protopia, a biological tool that offers a way of searching for proteins and their interactions in different Protein Interaction Web Databases, as a part of a multidisciplinary initiative of our institution for the integration of biological data .

**Results:**

The tool accesses the different Databases (at present, the free version of Transfac, DIP, Hprd, Int-Act and iHop), and results are expressed with biological protein names or databases codes and can be depicted as a vector or a matrix. They can be represented and handled interactively as an organic graph. Comparison among databases is carried out using the Uniprot codes annotated for each protein.

**Conclusion:**

The tool locates and integrates the current information stored in the aforementioned databases, and redundancies among them are detected. Results are compatible with the most important network analysers, so that they can be compared and analysed by other world-wide known tools and platforms. The visualization possibilities help to attain this goal and they are especially interesting for handling multiple-step or complex networks.

## Background

A living organism is an open system that is constantly exchanging chemical compounds, energy and information with its environment. This exchange involves a large number of elements (molecules) related to each other in a dynamic hierarchical and modular manner. Modules can be identified from the analysis of the interaction patterns. At a molecular level, interacting networks include protein-protein interactions, metabolic pathways, and the different biosignalling pathways controlling intercellular cross-talk and the regulation of gene expression [1,2]. Thus, protein-protein interactions can be considered as the basic skeleton for living organism self-organization and homeostasis [3]. Consequently, understanding the structural data concerning network skeletons, as well as its one-to-one element interactions, is essential (though just beginning) for effective progress in the characterization of biological complex systems and in understanding the pathological consequences of alterations in the properties of a given node (protein). In fact, molecular interaction networks are widely studied to reveal the complex roles played by gene products and cellular environments in biological and pathological processes [4-6].

When a new interactome is built with current information integration tools, nodes represent macromolecules and connecting segments represent specific interactions. Nodes are associated with additional information about the genes/proteins; for instance, chromosome number and gene location, intracellular location of the protein, and codes for locations in different Data Banks and ontologies. All this information is extensively stored in several data warehouses (Expasy, Gene Bank, Uniprot, or Gene Ontology, among others) [7-10].

Multiple sources of information on protein to protein interaction exist. They have usually been analyzed individually by genetic, biochemical and biophysical techniques. Recently, however, new methods have been developed for high-throughput macromolecular interaction analysis, including both experimental and biocomputational approaches. The high speed of new data generation has caused a problem, namely standardization of its notation in database(s) [11]. In the most significant interaction databases, the information provided by these methods is stored, curated and commonly linked to node data warehouses, but many details are not always fully or clearly specified (for instance, type of interaction, experimental conditions, etc). This lack of precision in interaction description is a major problem of the high-throughput data repositories. Further efforts are therefore essential to improve the quality of the protein-protein interaction databases to reach a level of confidence on crude networks, to save manual curation efforts and, consequently, to gain efficiency in inferring useful biological information from the interactome graphs. In addition, intersection and overlapping among these databases is limited and, therefore, the information is complementary in many cases. Thus, information should be unified to increase and to improve knowledge about interaction networks. In this way, several databases are working to integrate all this information on flexible platforms: BiologicalNetwork, Agile Protein Interaction Data-Analyzer (APID), KEGG or Protein Launge [12-14]. Finally, even partial interactomes on a given intracellular process often contains hundreds of nodes which are difficult to manage during analysis. Consequently, efforts to improve result visualization and compatibility among different platforms would also be helpful.

In this paper, we present a tool called Protopia for searching for and integrating protein-protein interactions and the information about them contained in five different Protein Interaction Web Databases. It can be useful as a friendly search interface among different databases, as a validator of redundant information, as a network visualization tool and also as an export tool to SBML [15].

## Methods

**Figure 1:**
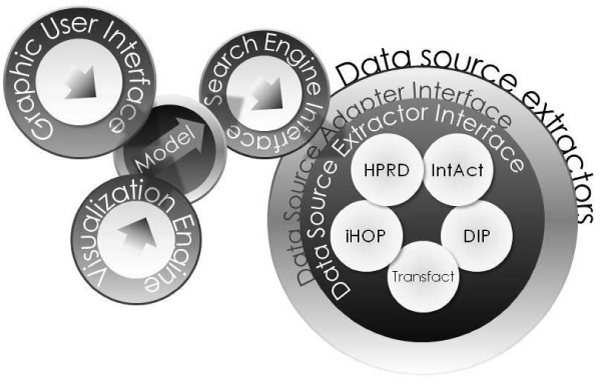
**Protopia's architecture**. The diagram of Protopia's architecture shows the decoupling between all their components.

Protopia is a modularized application, with separate functionalities providing a high degree of reusability and error correction. There are several main modules (Figure 1) that work together but are built separately with a different functionality: *Graphic User Interface*, *Model*, *Visualization Engine*, *Search Engine Interface*, *Data Source Adapter Interface *and *Data Source Extractor Interface*. Protopia has been implemented in Java because it makes it possible to manage all required software patterns and also provides Operative System independence. Protopia components have been designed to increase reusability and the possibility of including new databases when required. The description of these components is shown below:

• *The Graphic User Interface*: A user interface is provided to use and to manage all the capabilities of Protopia in an easy manner.

• *Model*: It is the component that manages all the other components, and makes cooperation amongst them possible.

• *Visualization Engine*: It provides diverse representations of the located protein to protein interactions. Two kinds of text representations (vector and matrix), and two kinds of graph representations (hierarchical and an organic hypergraph) have been implemented using a hypergraph [16] library and the Graphviz [17] library.

• *Search Engine Interface*: It uses the *Data Source Adapter Interface *of every *Data Service *implemented to perform the specific search among the data sources included in Protopia.

• *Data Source Adapter Interface*: An abstract interface defined to be implemented by a custom data source extractor in order to be able to both communicate and cooperate with the *Search Engine Interface*. It describes some basic operations to implement the service.

• *Data Source Extractor Interface*: An abstract interface defined to be implemented by a custom data source extractor in order to be able to transform the original data to a common data structure usable by the *Search Engine Interface*.

### Data source extractors

**Figure 2:**
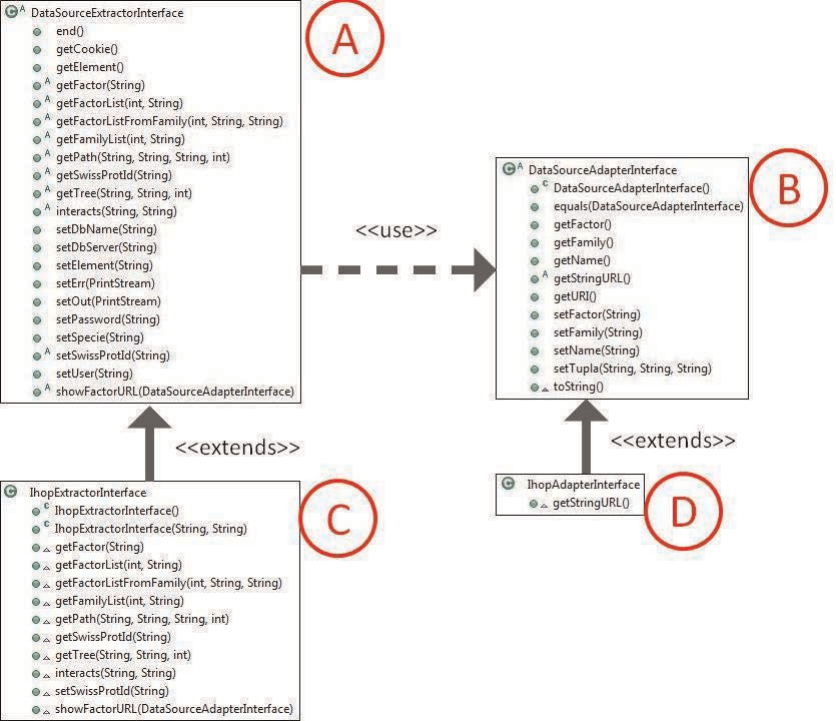
**Data source extraction scheme**. Abstract classes A and B define the operations required and implement the functionality between them. Scheme shows the class diagram which represents the extraction's implementation in IHOP. For other sources classes C and D are different.

The data source extractors are the components capable of extracting and transforming the protein interaction information. They implement the Data Source Adapter Interface and the Data Source Extractor Interface required to make them compatible with Protopia (Figure 2).

This project has been implemented with five data source extractors from five main Protein Web Databases (the free version of Transfac, DIP, Hprd, Int-Act and iHop) by using an access via HTTP. It was achieved by implementing parses among the HTTP text. Thus, our tool is able to use an abstract interface that defines the extraction and adaptation of the data by applying a usual data extraction method (via HTTP parse), but it can also be deployed by using direct access to databases (via FTP), web services, and other available data access.

When a data source extractor is needed, it is easy to provide a new one by extending two java classes, one to define the URLs and characteristics (Data Source Adapter Interface) and the other one to define the way to obtain the different nodes and interactions of the graphs (Data Source Extractor Interface). In the same way, it is possible to build custom data source extractors for a desirable data source without the need to recompile the source code, in other words simply by packaging those two implemented classes, by creating a simple jar, and by copying it in an interface folder. Protopia automatically detects the changes in the folder and loads the new data source extractor.

### Data representation and interoperability

Most efforts in Protopia have been directed to providing useful and friendly protein interaction graph representations. In Protein to Protein interaction graph visualization a perfect visualization method does not exist, but there are applications that achieve it with a moderate to high degree of satisfaction. That is the case of ProViz, iPfam or Cytoscape [18-20]. Since protein to protein graphs tend to be very big, you have to choose either to have a global representation of the graph or to have a detailed representation of a part of the graph. For these reasons, we consider to improve two different visualization ways that full equip Protopia as a complete interaction viewer.

**Figure 3:**
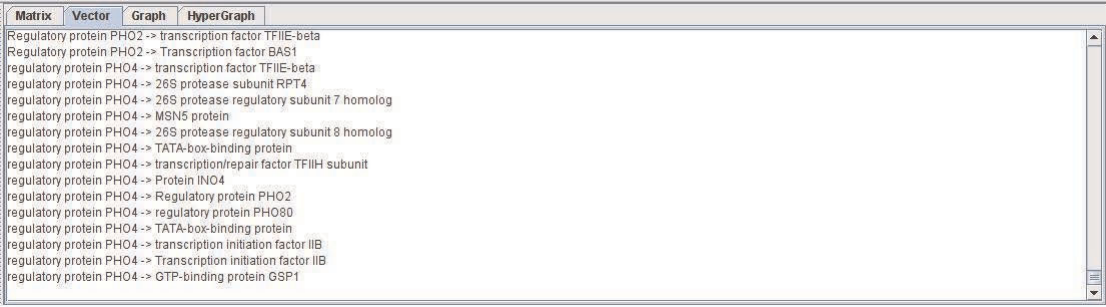
**Text vector implementation**. The figure shows the results obtained from DIP for P53 [DIP:368] [SwissProt:P04367]. This kind of data representation was thought to allow experimented biologist to obtain a file with plain text as target for a program input.

**Figure 4:**
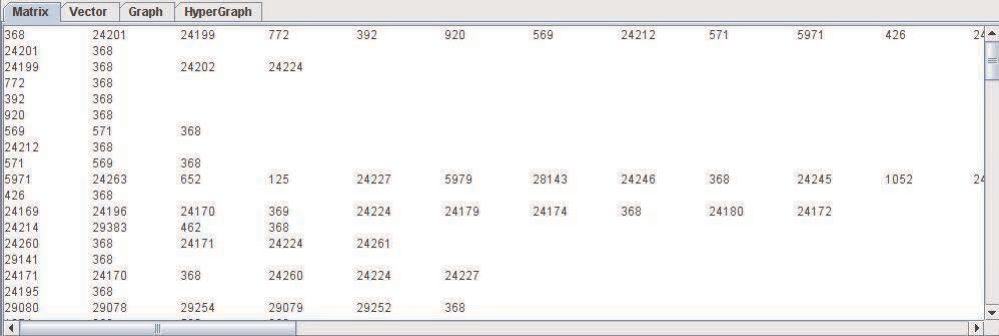
**Text matrix implementation**. The figure shows the results obtained from DIP for P53 [DIP:368] [SwissProt:P04367]. This kind of data representation was thought to simplify the view of interactions in text mode.

**Figure 5:**
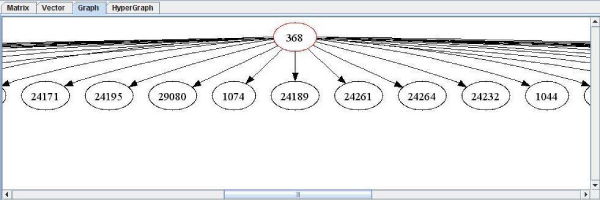
**Graphviz visual implementation**. The figure shows the results obtained from DIP for P53 [DIP:368] [SwissProt:P04367]. This kind of graph view had a hierarchical layout capability, used to see the interactions by his level.

**Figure 6:**
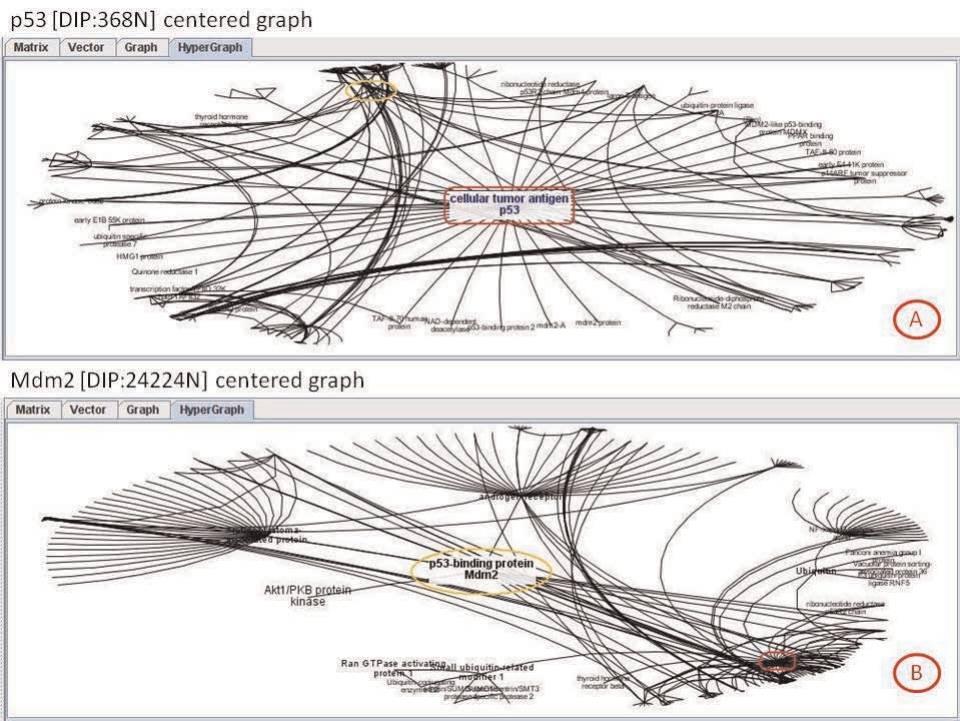
**Hypergraph visual implementation**. In A the view is centered over the P53 protein [DIP:368] [SwissProt:P04367]. In B the view is centered over the p53-binding protein Mdm2 [DIP:24224N] [SwissProt:Q00987].

Four different kinds of visualization have been implemented. In all cases proteins can be represented by either the abbreviated name or the code of the respective Data Bank:

• The first one is based on a text vector form (Figure 3) and the second one is based on a text matrix form (Figure 4). They have been implemented in text mode to provide a simple and low load mode that allows copying and pasting the results easily.

• The third one is based on the Graphviz [17] library, which provides a hierarchic and schematic point of view of the interaction graph and enables the user to analyse the graph in a global way. Implementation of the Graphviz view mode is simple and it only makes external calls to the Graphviz package (Figure 5).

• The fourth one has been implemented on the basis of an organic hypergraph library [16], which provides a fish eye view and makes it easier to analyse the net based on a main protein and its interacting neighbourhood (Figure 6). This visualization method enables the user to interact with the graph and change it (organically) to centre the view on the desired area, which is extremely useful for analysing a specific part of the graph. Implementation was carried out by extending the corresponding java classes to reach the desirable functionalities. Since our tool is able to manage up to more than one thousand nodes of a several-step network, this visualization is particularly convenient and helpful for complex network visualization and analysis.

On the other hand, an optimum system should be able to reach a high degree of integration with any information repository, as well as maximum interoperability among the different data analysis tools. In our system integration of a new Data source will be easily executed by following the same procedure described above. Reciprocally, the information collected by our tool can be recorded in a file, saving the interaction data graph in plain text mode or in the standard SBML format, making results compatible with many otherplatforms (for instance, Copasy, APID, etc), which is interesting for the comparative analysis of different networks.

### Detection of redundancies

An optimum system should be able to locate and unify the information on a given molecule stored under any of its identifiers and return a confidence value to the user for each located edge.

The increasing activity of the scientific community in applying technologies feeding protein-protein interaction information (for instance, bioinformatics predictions from HTP-technologies, 2-hybrids, pool-down, fluorescence correlation spectroscopy, fluorescence resonance energy transfer), followed by their notations in Data Banks, makes it likely that information on protein-protein interactions will grow exponentially in the short-term. It would be convenient that such a volume of information could be discriminated to select the most relevant in order to avoid an undesirable increase of background. Multiple facts should be taken into account for an accurate estimation of confidence; for instance, the method(s) used to deduce such an interaction (*in silico*, *in vitro *and/or *in vivo*). This information is often available in databases, but the different technologies and their values integrated in a "global confidence score algorithm" is not an easy task, and may be premature. In our opinion, such an algorithm should be reached by consensus and its cut-off should be selected by the user, which could be particularly important for studies of poorly-characterized interactomes.

At the present stage, with this goal in mind and taking into account the heterogeneous information captured by the different repositories and the dynamics of new inputs, a simple (but slanted) approach to the final objective could be provided by the quotient between the number of repositories-containing information on the interaction and the total number of queried databases. In our tool, this quotient is called "redundancy".

### Redundancy algorithm and values

**Figure 7:**
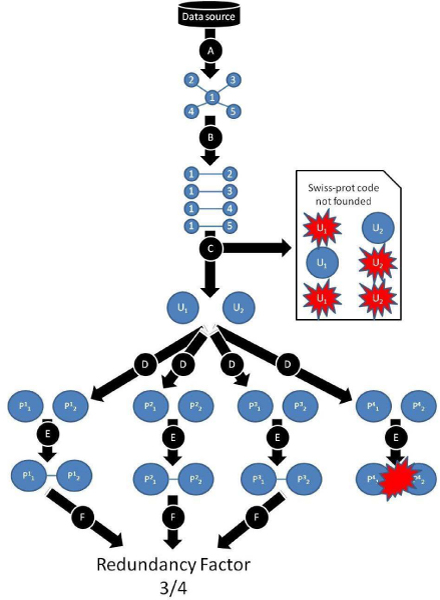
**Redundancy analysis algorithm scheme**. In step *A *a network is captured from a given database. In step *B*, the network is decomposed into its interaction pairs. In step *C*, Protopia looks for the SwissProt code of both proteins of every pair captured. In step *D*, the specific database code (for each of the other databases) for the SwissProt code is located. In step *E*, the interactions between the specific codes of the pairs are located in all the other databases. Finally, step *F *checks if the interaction has been found, and in this case a unit is added to the redundancy factor numerator.

The Protopia algorithm to look for redundancy is based on a simple but efficient search across all the available data sources (see Figure 7). The tool validates every pair of proteins this way: For every pair obtained from one of the data bases (for instance, DIP), we obtain the respective Swissprot codes (step C in Figure 7). Then, both protein codes are the parameter to search for the corresponding identifier code of every other data source (step D in Figure 7). Once both identifiers are available, an interaction between these two codes is performed in every data source. The original database where the interaction was first detected is not taken into account in the quotient.

Then, Protopia can validate those interactions searching among all the integrated databases, looking for those relationships in all the databases and retrieving the validation for the user. This is called the redundancy factor. Depending on the visualization mode, the results are expressed as quotients on the edges and/or by a three colour code for the edges (see *Example of Redundancy Value *Section). Thus, high redundancy (the darkest edges) will be considered when the numerator is as big as the denominator. It means that the interaction is redundantly defined in all the databases used in Protopia. No redundancy (the lightest edges) will be considered when the numerator is one. It means that the interaction is defined only in the original database and no matches have been obtained from searching in other databases. Medium redundancy will be considered in other cases. It means that the interaction is defined in some (more than one) of the databases used in Protopia.

### Example of redundancy value

This example shows an example of a single step search of a well known suppressor gene product, *p53*, described as a hub by several authors, including our own results [21,22]. In DIP, it is described as "cellular tumor antigen P53", with DIP code "368". For this protein, 37 interaction pairs were found.

**Figure 8:**
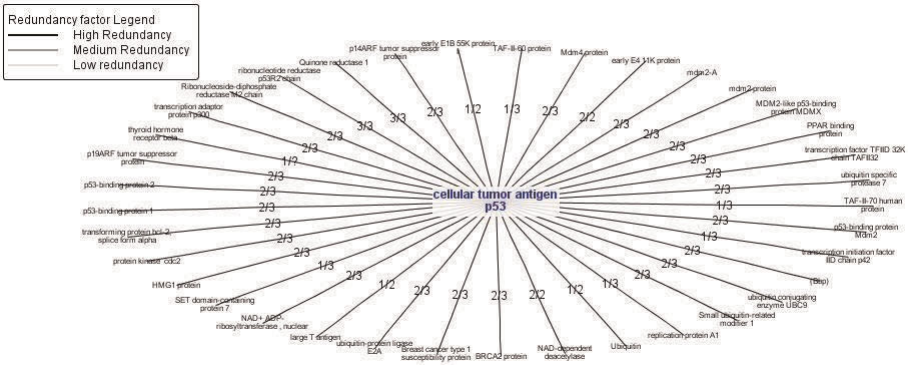
**Redundancy analysis example**. Redundancy analysis hypergraph of the "cellular tumor antigen" p53 network. The redundancy of ribonucleotide reductase p53R2 chain is the highest possible, and we will expect a high interaction probability.

The interaction redundancy hypergraph of the other data sources implemented in Protopia (Transfac Web, Intact, HPRP, IHOP) was analysed and is shown in Figure 8. It is obvious that one of the data sources could not locate or obtain the Swiss-Prot code of the protein P53 [Swiss-Prot: P04637] in their database, because the maximum denominator is 3 and the number of databases compared was four. For this reason we believe that is important to use Web services instead of HTTP access. However, most of the databases do not provide this kind of interface. Another reason for a lower redundancy is because we use Transfac Web that does not allow access to all its proteins in the free version.

In this example, only 62.16% of the protein interactions were redundant to some extent. Of this percentage, only 10.81% of the interactions are described in three of the databases, 40.54% are described in two of the databases, and 10.81% are described in just one of the databases.

## Results

**Figure 9:**
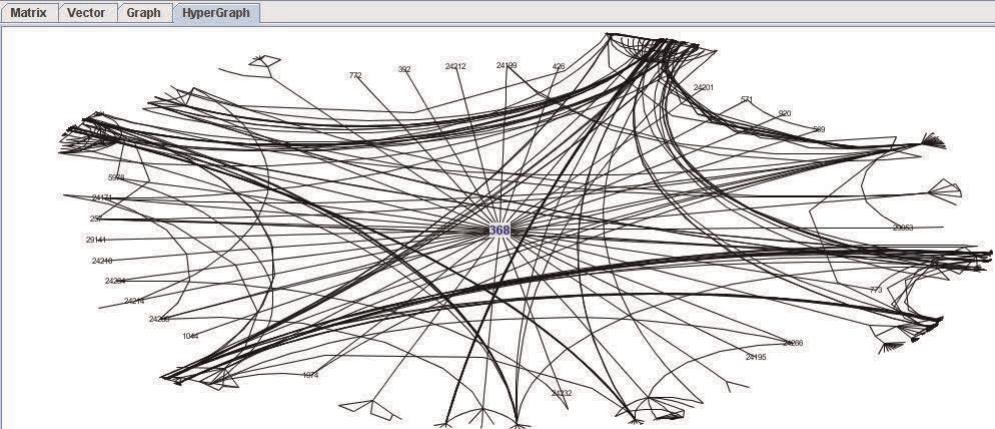
**Visualization**. 4-level-deep network visualization of p53 protein [DIP:368] [SwissProt:P04367] that involves a total number of 836 proteins and 1595 interactions. The Figure is centered on p53 and is represented by an interactive organic graph as explained below.

Protopia is one of the biocomputational tools implemented by the ASP project . This section will describe Protopia characteristics. Briefly, Protopia provides a configuration tool for selecting the database(s) used to retrieve information. Once the target database(s) has been selected, users can search by either the name or the code of the desired protein, and it retrieves a list of proteins from which to choose the required one. Once the protein is chosen, the tool will search its interactions through the selected database(s), and returns a collection of proteins and their interactions. The result of a search can be visualized in three different ways: a vector, a matrix, and a graph (Figure 9); results can also be saved in text formats or in SBML format. It is also possible to use logic operators to compare the interactions between two specific proteins, and to search all the paths between two proteins. By default, the search ratio has just one level of interactions, but it is a configurable option and the tool can show interactions up to a 10-level deep tree. This tree-graph is a way of showing protein interactions quickly and efficiently, but it is also an interactive way of visiting the web page where the tool has queried to obtain the information. The visualization engine is an interactive and navigable graph that offers a nice visualization of a specific zone of the graph.

Protopia actually integrates five Web Protein Databases: the free version of Transfac, DIP, Hprd, Int-Act and iHop [23-27]. These are five of the most important interaction protein databases existing at present, and we search all the data through queries directly to the Web Server.

As an example of use the well known protein p53 has been presented in previous sections. In summary, looking for its interactions in Transfac we notice that there are 36 interactions with other proteins (Figure 5). But, users could be interested in looking for the interactions of those 36 proteins with other proteins. So, the interaction graph is expanded to the second level (Figure 6). This graph can be also validated with respect to the other databases. So, the user obtains the redundancy factor as measure to quantify the real possible probability of interaction, if it is desirable to filter the huge quantity of interactions on an automatic way to allow the expert to study only the more probably interaction literature (Figure 8).

## Discussion

Protopia deals with integration problems caused by the heterogeneity of Biological databases. For instance, there is no universal identification code for proteins accepted by the whole scientific community. Protopia tries to validate interactions using the Swissprot code which is usually available in most protein data files, matching them with this code which is also available in Expasy. However, if for example the user is using the HPRD engine, the Swissprot code of the p53 regulated apoptosis inducing protein 1 is not available (it is possible to visualize the problem by visiting the page and looking for the empty Swiss-Prot field in this HPRD web link: ). Consequently, the redundancy quotients of any interaction involving p53 are lacking the information coming from HPRD. This problem is solved by analyzing the known names or synonyms (or even the sequence) when the Swissprot code is not available (due to an annotation error or because the protein is not identified by the Uniprot).

Protopia scores its results on the basis of the redundancies in the interaction information among the different repositories. Obtaining a higher redundancy level between PPI databases is only a matter of time. However, for some species like humans [28] a low redundancy level still exists among the different databases. Currently, this inconvenience hampers the accuracy of any attempt geared at computer-assisted validation of the results. Also, we are not always able to find via HTTP the protein that belongs to a Swiss-Prot code, because sometimes it is impossible to query some parameters this way and the redundancy factor could be lower because of this lack, this is the reason we claim to databases to offer a best way to access data, like web services. In our opinion, it is time to reach consensus on a more unified way of making new annotations and to have a score algorithm accepted by the scientific community in order to help information integration and interchange and to obtain maximum benefits from the huge experimental efforts that are being carried out at present. To evaluate the quality of the interaction, we agree with the need to standardize and to score the annotations regarding experimental methods, sources, etc used in the described interaction, also claimed by other groups [1].

The access to data sources has been implemented via HTTP, which could seem more expensive in time and data transfer than other tools working on downloaded information. In turn, our tool allows an easy online way to access current data. Thus, the information obtained is always up-to-date. In our approach, information location problems will be solved when most of the public databases develop a web service that allows us to ask for just a given protein.

## Conclusion

Protopia has become a useful tool to search for, visualize and validate protein interactions, by providing an intuitive user interface and useful configurability. This tool provides multiple ways of representing the results, being useful for researchers to quickly and efficiently find protein interactions and long-distance protein-protein relationships. One of Protopia's best features is that it is designed to access online data. Therefore, it is unnecessary to keep large quantities of data (and periodically download the full database). Only a little cache disk space is maintained to provide quicker access to the interaction information. The ease in implementing a new data source extractor interface adds functionality and new sources to compare interactions. The possibility of exporting the interactions to the standard SBML format enables the reuse of the interaction graph with other programs and tools. Thus, Protopia is not just an end-user application; it can also be used as a protein to protein interaction extractor capable of saving the selected proteins and interactions in a SBML file for subsequent analysis and manipulation.

Current databases offer a different level of curation, annotation, number of proteins, and number of interactions. We intend to continue working towards taking all this information (and all future information) to offer an improved interaction analysis method and tool able to take into account new filters, as if they were the tissues of an organism, and the experimental technology used to obtain the information. In fact, we believe that in the future (when enough information will have been extracted and annotated) the ideal tool should be able to detect and compare intercellular differences [29]. In the short/medium-term (over the next 1–2 years), we are planning to develop a CellDesigner plug-in that incorporates the two Protopia visualizations, as well as another plug-in that uses different access databanks to evaluate and validate PPIs with the new ideas proposed.

It is accepted that this kind of biocomputational effort and tool is essential for the advance of knowledge in Systems Biology. Analyzing protein interaction networks using structural information is one of these approaches [1]. In particular, our group requires them as part of a dual in *silico*-experimental strategy to study different biomedical problems [30]. Cancer, inflammation/immunity problems and orphan diseases are several of these biomedical problems for which network characterizations could reveal important emergent properties to understand and control the frequently dramatic systemic consequences of alterations in just a simple metabolic element. Important efforts are being made in cancer and inflammation/immunity problems [31-33] to advance in the knowledge and intervention possibilities of these pathologies. Nevertheless, it is for orphan/rare diseases, many of them caused by mutations, for which in *silico *approaches can provide the maximum degree of information, mostly due to the scarce number of human samples, as a result of which advance is only possible by using experimental approaches [34,35]. Due to the complexity of the human organism and different cell and tissue-specificities, it is almost impossible to obtain a complete picture of the different syndromes at the human level just by integrating the information provided by studies carried out on the isolated protein elements affected in each case, or by biochemical results obtained with cultured cell type models. This task requires systemic approaches, including the development of new in *silico *tools not only to integrate information, but also to filter and to organize the information obtained from many thousands of experimental molecular, biochemical and cellular studies in an automated way.

Characterization of protein networks, as well as formalization (mathematic modelling) of the metabolic behaviour could help the scientific community in searching for biochemical answers at the systemic level for many pathologies, including orphan diseases. It could allow us to predict the consequences of a given alteration in one of the protein/gene elements involved in a metabolic pathway, taking into account the cell specificities of the different human tissues and cell types. In fact, due to the differential expression patterns of the different cell types, a similar genetic alteration can be compensated or can have little importance in the correct function of one cell type, whereas it could have important consequences in the function of another one. This approach can also be useful to obtain emergent information on how to transmit the consequences of a molecular alteration to other apparently long-distance metabolic reactions of a given cell type, thus helping to understand (at least partially) the biochemical causes for the loss of homeostasis of patients. As evidenced in the acknowledgement section and affiliation, our group is engaged in different projects and networks (on cancer, inflammation, angiogenesis, and rare diseases) with the aim of contributing to a better understanding of the molecular causes or consequences of any of these pathologies and, consequently, to the design of new and more efficient intervention strategies.

## Availability and requirements

• **Project name**: Protopia, A protein-protein interaction tool.

• **Project home page**: 

• **Operating System**: All which supports Java.

• **Programming language**: Java.

• **Other requirements**: Java SE 6.0, Graphviz to visualize dot graphs.

• **License**: Creative Commons, 

• **Manual**: 

## Competing interests

The authors declare that they have no competing interests.

## Authors' contributions

ARC has designed the architecture of Protopia, drafted the manual and implemented together with IRM, AK and OC the tool. IND has implemented the extractors and drafted the manual. FJS and MAM have conceived the infrastructure, testing the tool, coordination and helped to draft the manual. JAM participate on the architecture's design, conceived the infrastructure, coordination, and helped to draft the manual.
